# Severe Combined Immunodeficiency—Classification, Microbiology Association and Treatment

**DOI:** 10.3390/microorganisms11061589

**Published:** 2023-06-15

**Authors:** Angel A. Justiz-Vaillant, Darren Gopaul, Patrick Eberechi Akpaka, Sachin Soodeen, Rodolfo Arozarena Fundora

**Affiliations:** 1 Department of Paraclinical Sciences, Faculty of Medical Sciences, The University of the West Indies, St. Augustine, Trinidad and Tobago; patrick.akpaka@sta.uwi.edu (P.E.A.); sachin.soodeen@my.uwi.edu (S.S.); 2 Department of Internal Medicine, Port of Spain General Hospital, The University of the West Indies, St. Augustine, Trinidad and Tobago; 3 Eric Williams Medical Sciences Complex, North Central Regional Health Authority, Champs Fleurs, Trinidad and Tobago; rodolfo.fundora@sta.uwi.edu; 4 Department of Clinical and Surgical Sciences, Faculty of Medical Sciences, The University of the West Indies, St. Augustine, Trinidad and Tobago

**Keywords:** microorganisms, severe combined immunodeficiency, infectious diseases, management, antibiotics, antifungals, HST

## Abstract

Severe combined immunodeficiency (SCID) is a primary inherited immunodeficiency disease that presents before the age of three months and can be fatal. It is usually due to opportunistic infections caused by bacteria, viruses, fungi, and protozoa resulting in a decrease in number and impairment in the function of T and B cells. Autosomal, X-linked, and sporadic forms exist. Evidence of recurrent opportunistic infections and lymphopenia very early in life should prompt immunological investigation and suspicion of this rare disorder. Adequate stem cell transplantation is the treatment of choice. This review aimed to provide a comprehensive approach to the microorganisms associated with severe combined immunodeficiency (SCID) and its management. We describe SCID as a syndrome and summarize the different microorganisms that affect children and how they can be investigated and treated.

## 1. Introduction

There are rare situations in which the immune system fails to mature at birth, and this can result in a primary immunodeficiency disease (PID) [1]. The immune system comprises the innate and adaptive immune responses. The innate response is the first line of defense against microorganisms. The adaptive immune response includes T-cell-mediated immunity, which destroys viruses and other intracellular microorganisms, and B-cell-mediated immunity, which eliminates bacteria and other extracellular microorganisms [2,3,4]. Of all the many PIDs described, severe combined immunodeficiency disease (SCID) is the best studied. It is considered a pediatric emergency in children [5]. The affected infants have a severely weakened immune system, leading to their inability to effectively protect against infection, even by the least pathogenic microorganisms [2,6]. In this review, we describe the different types of SCID, along with the different microorganisms that affect patients, and how they are detected and treated.

SCID, or “the bubble boy disease”, is a rare disorder in which multiple genes involved in the development and function of various immune cells undergo mutation [7]. This condition affects both the adaptive and innate immune systems, often resulting in fatal complications within the first two years of life unless treated with hematopoietic stem cell therapy (HSCT), or gene therapy [8,9]. In the United States, SCID was added to the Recommended Uniform Screening Panel (RUSP) in 2010, and newborns are now screened for this highly fatal disease [10].

SCID can be defined as typical, atypical/leaky, Variant, or Omenn Syndrome [11,12,13]. “According to the European Society for Immunodeficiencies (ESID). Typical SCID is defined as a patient with: (a) mutation(s) in a gene associated with a typical SCID phenotype; or, (b) presentation with severe or opportunistic infections, persistent diarrhea and failure to thrive, in the presence of low (300/μL) or absent CD3+ or CD4+ or CD8+ T cells, reduced naive CD4+ (CD3+CD4+CD45RA+) and/or CD8+ (CD3+CD8+CD45RA+) T cells, elevated γδ T cells, absence of proliferative responses to mitogens, defined as proliferative response to phytohemagglutinin (PHA) lower than 10% of the control subject; or (c) T cells of maternal origin present. The most common types typical SCID often include X-linked SCID, adenosine deaminase deficiency SCID, RAG-1/RAG-2 deficiency, and IL7R SCID” [14].

More than a dozen genes are involved in the pathogenesis of SCID [15]. SCID is most commonly inherited in an X-linked recessive or autosomal recessive manner [2,9,16]. Although the diagnosis of SCID is usually made by flow cytometry, genetic testing is often needed for genetic counseling and prognostication [15]. However, early diagnosis and treatment could be missed or even delayed, because although SCID is often caused by many genetic factors, over 80% of cases of SCID are sporadic, with no known family history of congenital immunodeficiencies [17,18].

Atypical SCID is characterized by CD3+ > 300 cells/μL and reduced, but detectable, proliferative response to PHA (>10 < 30% of the control) [14]. It is also sometimes referred to as “leaky SCID” [19]. Variant SCID is diagnosed in cases with no known gene defect and a persistence of 300–1500 T cells/L with impaired function [20]. 

## 2. SCID Classification and Features

### 2.1. X-Linked SCID

In this disease, the immune system makes very few T cells and natural killer cells (NK cells). Approximately 50% of SCID cases are X-linked, and this represent the most common form of SCID [16]. It is caused by mutations in the gene encoding the γ_c_ chain of the interleukin (IL)-2 receptor. This receptor is essential for thymic Treg development, and regulation of T-reg homeostasis and suppression. Without these cells, infection occurs frequently [21]. It primarily affects males.

### 2.2. Adenosine Deaminase Deficiency

This is the second most common form of SCID, accounting for 15% of all cases [22]. In this particular disorder, there is a deficiency of the adenosine deaminase (ADA) enzyme, which mediates the conversion of adenosine into inosine and subsequently deoxyadenosine into deoxyinosine [23,24], leading to an intracellular buildup of deoxyadenosine. Deoxyadenosine triphosphate (dATP) is a toxic metabolite of deoxyadenosine and is particularly toxic to lymphoid precursors. Consequently, ADA deficiency is characterized by lymphopenia. Lack of ADA enzyme leads to neurological problems such as hearing and visual impairment, cognitive problems, and movement disorders [6,25].

### 2.3. RAG-1 and RAG-2 Deficiency SCID

This is the third most common form of SCID, as it presents with mutations in Recombination activation genes 1 and 2 (RAG-1 and RAG-2) [9,26]. The RAGs work as a multi-subunit complex to cleave double-strand deoxynucleic acid (dsDNA) molecules between the antigen-receptor-coding segment and flanking recombination signal sequence (RSS), as they initiate V(D)J recombination shuffling DNA proteins, which are then expressed on the surface coding for specific antigens [27]. Without these enzymes, T cell receptor development fails, resulting in abnormal T cells, leading to the many infectious complications [2].

### 2.4. IL-7R Deficiency SCID

Hepatocyte growth factor and interlukin-7 (IL7) form a heterodimer that functions as a pre-pro B-cell growth-stimulating factor [28,29,30]. It has also been found to be one of the co-factors in T cell receptor beta (V(D)J rearrangement for the development of T lymphocytes. This is the fourth most common type of SCID. Infants with such a disorder have few or no T cells or B cells; however, because of the lack of T lymphocytes, B lymphocytes are not be able to undergo somatic hypermutation and class switching [30,31].

### 2.5. Leaky SCID

Leaky SCID occurs when a child has signs and symptoms quite similar to typical SCID. However, the T cell counts are not low enough to warrant classifying the disease as typical SCID [32,33]. It is named “leaky” because a good amount of T cells “leak” through and a normal count of T cells appear in the individual’s blood [32]. However, these T cells are unable to combat infections. In leaky SCID, T cells can exhibit autoimmune phenomena, because they become over-activated against organs and tissues, and hence cause the body to attack itself. It is characterized by clinical features such as itchy skin, hair loss, red skin, weakness, swollen lymph nodes, hepatomegaly, splenomegaly, and diarrhea. It can also cause thyroid problems and anemia [24]. Children with leaky SCID might develop different types of gene mutations in the same genes similar to an individual with typical SCID, including deficiencies of RAG-1 and RAG-2 genes. Sometimes, these children are misdiagnosed, and the disease is only discovered when they are older, even into adulthood [34]. It can also be observed that, in leaky SCID, the gene mutation allows a normal or increased T cell count, which affects the immune system.

### 2.6. Omenn Syndrome

Omenn Syndrome (OS) is caused by gene mutations resulting in high numbers of defective T cells but no defects in B cell and natural killer (NK) cells. The defect in the T cells causes severe defects in the child’s immune system. It is an extremely serious autosomal recessive inherited T+ or T++ SCID deficiency [35,36].

Omenn Syndrome can manifest as SCID or occur on its own. Genetic mutations that can cause OS include those in the genes encoding for adenosine deaminase deficiency, RAG-1, RAG-2, Artemis, and DNA ligase 4. Infants with OS suffer from autoimmune diseases in which the body attacks itself and any defective immune system components. Symptoms of this immunodeficiency include early onset of a seborrheic pruritic skin eruption, hair loss, lymphadenopathy, splenomegaly, and hepatomegaly. Eosinophilia is present and the serum IgE is always elevated [23]. OS was first reported as a distinct form of SCID. Unlike typical SCID, patients with OS have a high mortality rate due to several opportunistic infections. An important aspect of its diagnostic workout is a timely microbiological and histological examination of skin biopsy. 

### 2.7. CD3 Complex Component Deficiency SCID

The CD3 complex is known as a T cell pan marker. The CD3 complex plays an essential role in cell signaling or cell communication down to the nucleus, which is initiated by antigen binding. This is because of the multiple alpha, beta, gamma, delta, epsilon, and zeta transmembrane chains that cause downstream cell signaling to the nucleus, consequently allowing for cytokine formation and release. There are three subtypes: CD3D, CD3E, and CD247/CD3Z. This type of disorder is caused by mutations in these CD3-encoding genes which subsequently result in damage to T cells [37].

### 2.8. JAK3 Deficiency SCID

The Janus kinase 3 gene works synonymously with the interleukin 2 receptor gene (IL2RG) for interleukin 2, which promotes the growth of T lymphocytes (helper, cytotoxic, and regulatory) and natural killer cells. Due to this deficiency, patients with JAK3 deficiency SCID show very similar attributes to patients presenting with lymphopenia. However, since JAK3 is not located on the X chromosome, both male and female infants can be affected [38,39].

### 2.9. Other Forms of SCID

Other forms of SCID include IL-2 α-chain deficiency synthesis [40], surface receptor/transduction defects [41], and defective T cell receptor epsilon chain. Sometimes, the children present with autoimmunity only, including vitiligo, autoimmune hemolytic anemia, autoimmune enteropathy, and Hashimoto’s thyroiditis. Additionally, SCID symptoms have been reported in autoimmune hepatitis, Evans syndrome, and nephrotic syndrome in a few cases [42]. ZAP-70 deficiency with CD8 protein absence causes a SCID syndrome characterized by CD4+ T cell circulation not responding in vivo to TCR-mediated stimuli [43]. CD3 gamma subunit proteins have also been described in rare patients [44]. Bare lymphocyte syndrome is characterized by the lack of human leukocyte antigens 1 or 2 in immuno-deficient children and opportunistic infections by low-virulence microorganisms. It can be a combined primary immunodeficiency causing an impairment of both the humoral and cell-mediated immune responses [45,46], short-limbed dwarfism [47], which is a SCID variant, as well as Nezelof’s syndrome, which is a combined immunodeficiency with the present of immunoglobulins that presents after the child reaches five years of age [48]. Another variant of SCID is Griscelli’s syndrome, which is seen in patients with fine silvery hair, enlarged liver, and lymphadenopathy [49]. Th relatively common OKT4 epitope deficiency is identified by the absence of reactivity by the OKT4 monoclonal antibody to CD4+ T cells. The said epitope has been found to be polymorphic in Black, White, and Japanese populations. These patients are clinically characterized by mild susceptibility to infections [50].

## 3. Microorganisms Affecting SCID Patients

### 3.1. Viruses

Opportunistic viral infections, e.g., with cytomegalovirus, Epstein–Barr virus, adenovirus, enterovirus, herpes simplex virus, respiratory syncytial virus, rotavirus, and parainfluenza virus, can cause severe disseminated infections in SCID patients and can be fatal if left undiagnosed or untreated [51,52,53]. Cytomegalovirus (CMV) has been found to be excreted in breastmilk, and breastfeeding should not be advised for SCID patients unless the mother is found to be CMV-antibody-negative [15].

Infection with *Adenovirus* can manifest as ocular, respiratory, gastrointestinal, or hepatic diseases in immunocompetent patients and is often mild and self-limiting [54]. However, in patients with SCID, adenovirus may produce severe and prolonged viral pneumonia, bronchiolitis, hepatitis, or gastroenteritis, with a potentially fatal outcome [55].

Rotavirus is the leading cause of severe gastroenteritis in children, and vaccination is the mainstay of prevention [56]. However, the live rotavirus vaccine has been found to cause severe diarrhea in children with SCID and should therefore be avoided [57]. Epstein–Barr virus infections affect over 95% of the human population at some point in their lives, but are usually asymptomatic [58]. Symptomatic infections in adolescents may result in infectious mononucleosis characterized by fever, sore throat, splenomegaly, and lymphadenopathy. The virus typically attacks B cells; therefore, SCID patients with impaired or absent B cells are at an increased risk of EBV-associated lymphomas as a result of persistent viremia and lymphoproliferation [59].

Parvovirus B-19 is a common infection in rapidly dividing erythroid progenitor cells, with children being the main source of infection [60]. Immunocompetent host infections can be asymptomatic or symptomatic, and include erythema infectiosum, arthropathy, anemia, thrombocytopenia, hepatitis, and myocarditis. In immunocompromised hosts, infection with Parvovirus B-19, chronic red cell aplasia, acute lymphoblastic leukemia (ALL), and virus-associated hemophagocytic syndrome (VAHS) [61].

Varicella-zoster virus (VZV) infection occurs primarily via respiratory inoculation and establishes lifetime latency in the sensory ganglia of immunocompetent patients [62]. Immunocompromised patients are at an increased risk of complications, such as reactivation, herpes zoster, retinal necrosis, and even death [62]. Worldwide vaccination via live VZV vaccines has prevented many of the complications of VZV infection [63]; however, vaccination in SCID patients has been associated with disseminated infection [64] including vaccine-associated pneumonia [65], and should therefore be avoided.

### 3.2. Bacteria

Recurrent sinopulmonary infections are characteristic of primary immunodeficiencies such as SCID, and can result in severe complications including lung abscess, empyema, and pneumatocele. The bacterial causes of pneumonia include *Staphylococcus aureus*, *Pseudomonas* spp., *Mycobacterium bovis*, and other atypical mycobacteria [66]. Clinical imaging provides an important diagnostic clue in acute pulmonary infections in children with primary immunodeficiencies, as they often lack a thymic shadow [67].

Clinical manifestations of SCID include gastrointestinal infections, chronic diarrhea, and failure to thrive. Gram-positive bacteria, such as *Staphylococcus aureus*, and Gram-negative bacteria such as *Klebsiella pneumoniae, Pseudomonas aeruginosa*, *Burkholderia,* and *Chryseobacterium*, are also commonly implicated [21]. SCID patients who lack immunoglobulins are at constant risk of recurrent infections with encapsulated bacteria [68].

Omenn syndrome is an autosomal recessive form of SCID that is usually T-B-NK+ and is highly fatal owing to recurrent opportunistic infections [36,69]. Skin sepsis is observed in patients with Omenn syndrome. Skin sepsis in Omenn syndrome can occur due to colonization by bacteria such as *Staphylococcus aureus*, *Streptococcus pyogenes*, enterococcus, and Gram-negative bacteria such as Pseudomonas species [36,70,71]. Cutaneous manifestations of bacterial infections include recurrent and life-threatening skin abscesses, folliculitis, impetigo, and furunculosis [72]. Survival rarely exceeds several months after birth in the absence of curative treatment.

### 3.3. Fungi

Invasive fungal infections (IFI) rarely occur in immunocompetent individuals and are more likely to occur in patients with primary immunodeficiencies. Opportunistic fungal infections seen in SCID are similar to those in patients with AIDS, and are usually caused by opportunistic fungi such as *Pneumocystis jirovecii*, *Histoplasma capsulatum*, and *Cryptococcus neoformans* [73]. *Pneumocystis jirovecii* pneumonia is the most common respiratory infection in SCID, and it is often co-infected with a respiratory virus [70]. Patients with SCID may be offered prophylactic treatment against *Pneumocystis jirovecii* to prevent fatal complications.

Patients with SCID are at increased risk of disseminated fungal infections, with invasive *Candida albicans* and *Aspergillus* being the most prominent microorganisms [74]. Other rare microorganisms implicated in SCID include *Acremonium* and *Pichia* [75,76]. Colonization of the skin, oropharynx, and gut by *Candida albicans* typically manifests as persistent oral thrush or diaper dermatitis progressing to diffuse skin involvement [75]. Hematopoietic stem cell transplantation is the definitive treatment for SCID, and fluconazole (3 mg/kg OD) is administered as prophylaxis against candidiasis and is generally well tolerated by the patients [77].

Invasive aspergillosis (IA) is a life-threatening condition in immunocompromised children. Infection is typically acquired in the community or via nosocomial infections caused by exposure to hospital construction, renovation, and air-conditioning systems [78]. Bronchopneumonia is the most common presentation of infection with Aspergillosis in SCID, and other primary immunodeficiencies [79]. Other clinical manifestations of invasive aspergillosis include pulmonary infarction, pulmonary thrombosis, and pleural effusion [80,81].

Cryptococcosis is a subacute or chronic systemic mycosis caused by *Cryptococcus neoformans* [82,83]. *Cryptococcus neoformans* is an opportunistic fungus that infects immunocompromised individuals. The respiratory tract is the primary portal of entry and has been found to be fatal because of overwhelming pneumonia in patients with SCID [82]. *Cryptococcus neoformans* was found in the skin lesions of a patient with SCID who presented with a maculopapular rash along with lobar consolidation. The treatment was refractory to medical management, but responsive to hematopoietic stem cell therapy [84].

### 3.4. Parasites

Parasitic infections are the dominant cause of gastrointestinal disease in patients with SCID. Protozoans, e.g., *Giardia duodenalis* or *Giradia intestinalis* (*Giardia lamblia*) and *Cryptosporidium* spp. are the most common protozoans affecting patients with SCID. Other implicated parasites include *Schistosoma* species, *Blastocystis hominis, Fasciola* species, and *Trichostrongylus* species [85]. The gastrointestinal (GI) tract is the largest lymphoid organ of the body [86]. The GI manifestations are the second most common manifestations of primary immunodeficiency disorders (PID) after pulmonary disease [87]. Gastrointestinal disorders, such as chronic diarrhea, malabsorption, and abdominal pain, are seen in as many as 50% of patients with primary immunodeficiencies [88]. *Giardia intestinalis* is a zoonotic protozoan parasite typically found in the small intestine of humans and various animals. Infections can be asymptomatic or cause mild diarrhea in immunocompetent patients but can cause severe and chronic diarrhea and malabsorption in immunocompromised patients [89,90,91].

*Cryptosporidium* species, especially *C. parvum*, can cause severe and chronic enteropathy by releasing proinflammatory cytokines such as interleukin-8 (IL-8) in intestinal epithelial cells in patients with primary immunodeficiencies [92,93]. Disseminated cryptosporidiosis can lead to biliary tract disease, pancreatitis, pulmonary disease, and stunted growth in patients with SCID [92]. Disseminated cryptosporidiosis leading to overwhelming sepsis and death has been observed in patients with SCID [94]. Although the International Agency for Research on Cancer (IARC) has not considered protozoans as carcinogens for humans [95], *Cryptosporidium* has been associated with colonic adenocarcinoma in SCID mice [96]; therefore, physicians should be aware of this possible complication and infection in SCID patients should be treated promptly.

Immunization with vaccinia virus and bacille Calmette–Guérin (BCG) vaccine is widely used in several countries and has been observed to exacerbate factors in patients with SCID. These vaccines can lead to disseminated, fatal infection, and must not be administered to patients with SCID [97].

## 4. Treatment of SCID

### 4.1. Hematopoietic Stem Cell Therapy (HSCT)

Hematopoietic stem cell transplantation (HSCT) is the recommended potentially curative treatment for SCID [28,98]. Although lifesaving, HSCT only partially restores immunity, as recovery is a dynamic process [99]. SCID patients who received transplants before the age of 3.5 months had the highest survival rates [100]. Therefore, the best outcomes for SCID newborns are achieved through early transplantation [29]. Adenosine deaminase conjugated with polyethylene glycol (PEG-ADA), an enzyme replacement medication, has been used to treat SCID in children with ADA deficiency with modest effectiveness, although it is not curative [6].

Infants diagnosed with SCID are often treated with hematopoietic stem cell therapy (HSCT), which is also known as bone marrow transplantation. This is, in fact, no easy medical task to perform, and takes a lot of time and preparation [2,6]. After a suitable donor is identified, hematopoietic stem cells are drawn and infused into infants with SCID. Hematopoietic stem cells are immature, and they then develop into red blood cells, white blood cells, and platelets. These cells then multiply over time, and immunity is consequently achieved; this is shown by the survival rate of the procedure, which ranges from 70–95% [101,102].

Factors that affect and influence the outcome of this transplantation method include:

(i) Age and clinical condition at the time of diagnosis. This method is best performed at an earlier time (age of 1–4 months). This time will disallow and mitigate the chances of opportunistic infections and failure to thrive [29].
(ii) Hematopoietic stem cell donor. It is very unlikely that the affected infant possesses HLA-matched siblings; therefore, locating HLA-compatible volunteers is time consuming, giving way to new and worsening infections or disorders.
(iii) Pre-treatment conditioning. Prior to HSCT, children may be subjected to conditioning with chemotherapy or to prepare the child’s body to receive new stem cells. Patients undergoing this treatment experience frequent defective B cell reconstitution, requiring lifelong immunoglobulin replacement therapy [2,6].

### 4.2. Gene Therapy

Gene therapy can be a successful treatment, particularly for X-linked SCID [103,104]. In this process, stem cells are drawn from the patient’s bone marrow, the normal gene is inserted using a carrier known as a vector, and the repaired cells are returned to the patient [2]. Early attempts to use gene therapy to treat X-linked SCID were successful in restoring T cell function in children [16], but approximately 25% of the children developed leukemia two to five years after [105]. The vectors employed in using gene therapy were proposed to inappropriately activate genes that regulate cell development, resulting in leukemia [26]. Modern gene therapy techniques often employ modified vectors that are more efficient and associated with less potential complications [106].

Artemis SCID gene therapy is now available for infants diagnosed with X-linked SCID. In this procedure the hematopoietic stem cells containing the mutated gene are extracted from the bone marrow or blood. This extracted gene is now sent to the lab where “correct” copies are made. This copy is now infused into a deactivated virus, which efficiently penetrates hematopoietic stem cells. After the virus penetrates the cell, the normal HSC of the patient mixes with the new copy and is allowed to be a part of hematopoietic stem cells. This corrected form of the cell is now allowed to be divided and placed in a cryogenic state. The infant then receives conditioning whether it is in [6,107] the form of chemotherapy or immunosuppressive agents, where the sample is then inserted via a simple IV infusion where corrected hematopoietic stem cells are able to spread throughout the body [29].

### 4.3. Enzyme Replacement Therapy (ERT)

Patients with adenosine deaminase deficiency (ADA) lack the vital enzyme adenosine deaminase; therefore, infants receive a weekly intramuscular injection of elapegademase containing adenosine deaminase. As simple and pain-free as this procedure sounds, it does not permanently cure SCID, but is merely a temporary step before a much more permanent procedure such as HSCT or gene therapy [2]. It has also been proven that using enzyme replacement therapy before stem cell therapy can actually enhance and increase the number of T lymphocytes, which results in a lower occurrence of infection until a definitive method is used [108,109].

### 4.4. Treatment of Infections

While Stem Cell Therapy (SCT) is the definitive treatment of SCID, the use of reverse isolation, that is, keeping the patient in a protected environment, avoiding live vaccines, therapeutic use of immunoglobulins, an early prophylactic use of antimicrobials, can help treat infections [110]. Early prophylactic antibiotic therapy is widely used in SCID treatment to reduce the frequency and severity of infections, especially bacterial sinopulmonary infections. Prophylactic antiviral and antifungal therapy are also warranted in SCID [111,112]. New antifungal agents have been developed over the last two decades, including lipid formulations of amphotericin B as liposomal amphotericin B, new azoles (voriconazole, posaconazole, and isavuconazole), and echinocandins (micafungin, caspofungin, and anidulafungin) [23].

## 5. Examples of Antibiotic Prophylaxis Regimens Used in Patients with Immunodeficiency

Prophylaxis with Sulfametoxazol-trimetoprim (TMP-SMX) while awaiting definitive SCT is aimed at addressing pneumocystic pneumonia (PCP), which is most commonly caused by *Pneumocystis jirovecii,* as shown in Table 1 [111,112,113,114,115]. Fluconazole is often administered to prevent mucocutaneous candidiasis, and acyclovir to prevent herpes simplex virus infection. Antifungals have been used to treat invasive pulmonary aspergillosis in patients with SCID [23] Oral valganciclovir is being used as an alternative to ganciclovir in immunocompromised children infected with CMV, including those with SCID [116]. Valacyclovir was used in experiments of the tropism of herpes simplex virus (HSV-1) for human sensory neurons infected in vivo using dorsal root ganglion xenografts maintained in mice with SCID [117].

## 6. Conclusions

This review could serve as a foundation for further mechanistic and clinical studies in understanding disease pathogenesis as well as the development of effective treatment strategies for patients with SCID. A prophylactic treatment with antibiotics is available, especially for *Pneumocystis jirovecii*. Patients with SCID, as a PID, can be affected by other microorganisms, including *Candida* spp., *Aspergillus* spp., and cytomegalovirus, which pose a threat to the life of neonates and children. However, hematopoietic stem cell transplantation is a curative treatment for SCID, and gene therapy promises excellent results for certain SCID variants in the future.

## Figures and Tables

**Table 1:** Antimicrobial treatment/prophylaxis of microorganisms implicated in Severe Combined Immunodeficiency Disease.

Microorganisms	Antimicrobial and Dosage	Reference
*Staphylococcus aureus*	Amoxicillin: Children: 10–20 mg/kg per day, single dose or divided into 2× (maximum: 875 mg/day). Adolescents and adults: 875 mg. Amoxicillin and clavulanate: Children: 20 mg/kg per day single dose or divided into 2× (maximum: 875 mg/day based on amoxicillin)	[111]
*Streptococcus species*	Amoxicillin and clavulanate: Children: 20 mg/kg per day single dose or divided into 2× (maximum: 875 mg/day based on amoxicillin). Adolescents and adults: 875 mg (based on amoxicillin)	[112]
*Mycoplasma* spp.	Azithromycin: Children: 5–10 mg/kg/po 3×/week (maximum: 250 mg). Adolescents and adults: 250 mg po 3×/week	[111]
Gram-negative spp.	Amoxicillin and clavulanate: Children: 20 mg/kg per day single dose or divided into 2× (maximum: 875 mg/day based on amoxicillin). Adolescents and adults: 875 mg (based on amoxicillin)	[112]
*Atypical mycobacterium*	Azithromycin: Children: 20 mg/kg/po 1×/week (maximum dose of 1200 mg/week; can be given up to 600 mg 2×/without causing nausea at high doses). Adolescents and adults: 1200 mg 1×/week (or 600 mg 2×/week in case of nausea)	[111]
*Pneumocystitis jirovecii*	Trimetroprime/sulphamethazole: 15–20 mg/kg iv or po for 14–21 days	[113,114]
*Aspergillus* spp.	Voriconazole: ≤50 kg: 8 mg/kg/oral dose 2×/day (maximum per dose: 350 mg). >50 kg: 4 mg/kg/po 2×/day (maximum per dose: 200 mg)	[111]
*Candida* spp.	Fluconazole: Children: 6 mg/kg orally daily (max 400 mg). Adolescents and adults: 400 mg orally daily	[112]
Herpes simplex virus	Acyclovir: Children < 40 kg: 600 mg oral dose 4×/day	[111]
Varicella zoster virus	Children > 40 kg: 800 mg oral dose 4×/day. Adults: 800 mg oral dose 2×/day	[112]
*Cytomegalovirus*	Gancyclovir: Children aged 1 month to 16 years: oral dose (mg) = 7 × body surface area × creatinine clearance. Adolescents ≥ 17 years and adults with normal renal function: 900 mg oral dose 1×/day.	[115]

## Data Availability

Not applicable.
